# Dual division of focal plane polarimeters-based collinear reflection Mueller matrix fast imaging microscope

**DOI:** 10.1117/1.JBO.27.8.086501

**Published:** 2022-08-22

**Authors:** Tongyu Huang, Ruoyu Meng, Jiawei Song, Tongjun Bu, Yuanhuan Zhu, Migao Li, Ran Liao, Hui Ma

**Affiliations:** a Tsinghua University, Shenzhen International Graduate School, Shenzhen, China; b Tsinghua University, Department of Biomedical Engineering, Beijing, China; c New York University, Department of Biomedical Engineering, New York, United States; d Tsinghua University, Department of Physics, Beijing, China; e Tsinghua University, Tsinghua–Berkeley Shenzhen Institute, Center for Precision and Healthcare, Shenzhen, China; f Guangdong Liss Optical Instrument Co., Ltd., Guangzhou, China

**Keywords:** polarization, DoFP polarimeter, Mueller matrix, microscope, dynamic process

## Abstract

**Significance:**

Reflection Mueller matrix imaging is suitable for characterizing the microstructure of bulk specimens and probing dynamic processes in living animals, there are always demands for speed and accuracy for such applications to avoid possible artifacts and reveal a sample’s intrinsic properties.

**Aim:**

To demonstrate a design of collinear reflection Mueller matrix fast imaging microscope based on dual division of focal plane (DoFP) polarimeters (DoFPs-CRMMM) which has high measurement speed and accuracy.

**Approach:**

In DoFPs-CRMMM, to improve the measurement speed, we applied the dual DoFP polarimeters design on the collinear reflection system for the first time to achieve fast imaging in about 2 s. To improve the measurement accuracy, we improved the double-pass eigenvalue calibration method (dp-ECM) by background light correction, and explored the optimization of the set of reference samples.

**Results:**

DoFPs-CRMMM was applied to measure the standard polarization samples and monitor the tissue optical clearing process of an artificial layered bulk tissue. Results show that the system has satisfactory performance which can capture the variation of polarization properties during the dynamic process.

**Conclusions:**

We present the establishment and demo application of DoFPs-CRMMM. The measurement speed can be further accelerated for potential applications in monitoring dynamic processes or living biomedical systems.

## 1 Introduction

Polarization imaging techniques are sensitive to the anisotropic microstructure of samples, therefore can be regarded as a potential tool in biomedical studies. Mueller matrix (MM) provides the comprehensive characterization of sample’s polarization properties. When considering the polarization detection for bulk biological tissues such as living organs, backscattering MM imaging is more applicable. In recent years, many applications of backscattering MM imaging in biomedical fields have emerged, including endoscopy,^1^^–^^4^ cancer diagnosis,^5^ urology pathology detection,^6^ and characterization of tissue optical clearing (TOC).^7^ Compared to the oblique incident illumination reflection MM imaging system,^8^ collinear reflection MM imaging can obtain a set of rotation invariant parameters which are not affected by the sample’s azimuth angles,^9^^,^^10^ and is therefore particularly suitable for probing the intrinsic polarization properties of the anisotropic samples. For polarization monitoring on living animals or dynamic processes, higher measurement speed and accuracy are always in demand.

There are many previous works on collinear reflection MM measurements, including point-measurement^11^^,^^12^ and imaging.^13^^–^^18^ We have reported a collinear reflection Mueller matrix microscope (CRMMM) based on dual rotating retarders (DRR-CRMMM),^19^ both the polarization state generator (PSG) and the polarization state analyzer (PSA) consist of a fixed linear polarizer and a rotatable phase retarder. According to the working principle of Fourier coefficients analysis,^20^ during each measurement, two-phase retarders rotate in steps of a fixed ratio of angles and 30 intensity images are acquired to reconstruct sample’s MM. Since the polarization modulation is based on division of time (DoT), the measurement is time-consuming (about 3 mins) and subjected to serious artifacts when measuring living samples or dynamic processes.

To perform fast polarization imaging, division of focal plane (DoFP) polarimeters have been widely used in recent years.^21^^,^^22^ DoFP polarimeter can measure the linear polarization states simultaneously by utilizing a pixelated micropolarizer array in front of the imaging sensor. When using a DoFP polarimeter and a variable phase retarder to take at least two acquisitions,^23^^,^^24^ or dual DoFP polarimeters and a fixed retarder for a single shot,^25^ complete Stokes vector measurement can also be realized.

In this paper, we demonstrate the implementation of the dual DoFP polarimeters-based DoFPs-CRMMM. To improve the measurement speed, DoFPs-CRMMM combines the dual DoFP polarimeters design and the collinear reflection measurement system and utilizes the fast polarization imaging ability of DoFP polarimeters to take a MM image in about 2 s. To improve the measurement accuracy, we improve the double-pass eigenvalue calibration method (dp-ECM)^13^ by background light correction and discuss the effect of the optimized reference samples set on the calibration performance to realize high accuracy MM imaging of bulk samples. After calibration, the measurement performance of DoFPs-CRMMM is validated by measuring standard polarization samples. To demonstrate the application potential, DoFPs-CRMMM is applied to fast polarization monitoring of the bulk samples during dynamic process of tissue optical clearing^26^ and prove its ability in biomedical research.

## 2 Materials and Methods

### 2.1 Experimental Setup and Mathematical Model

**Fig. 1:**
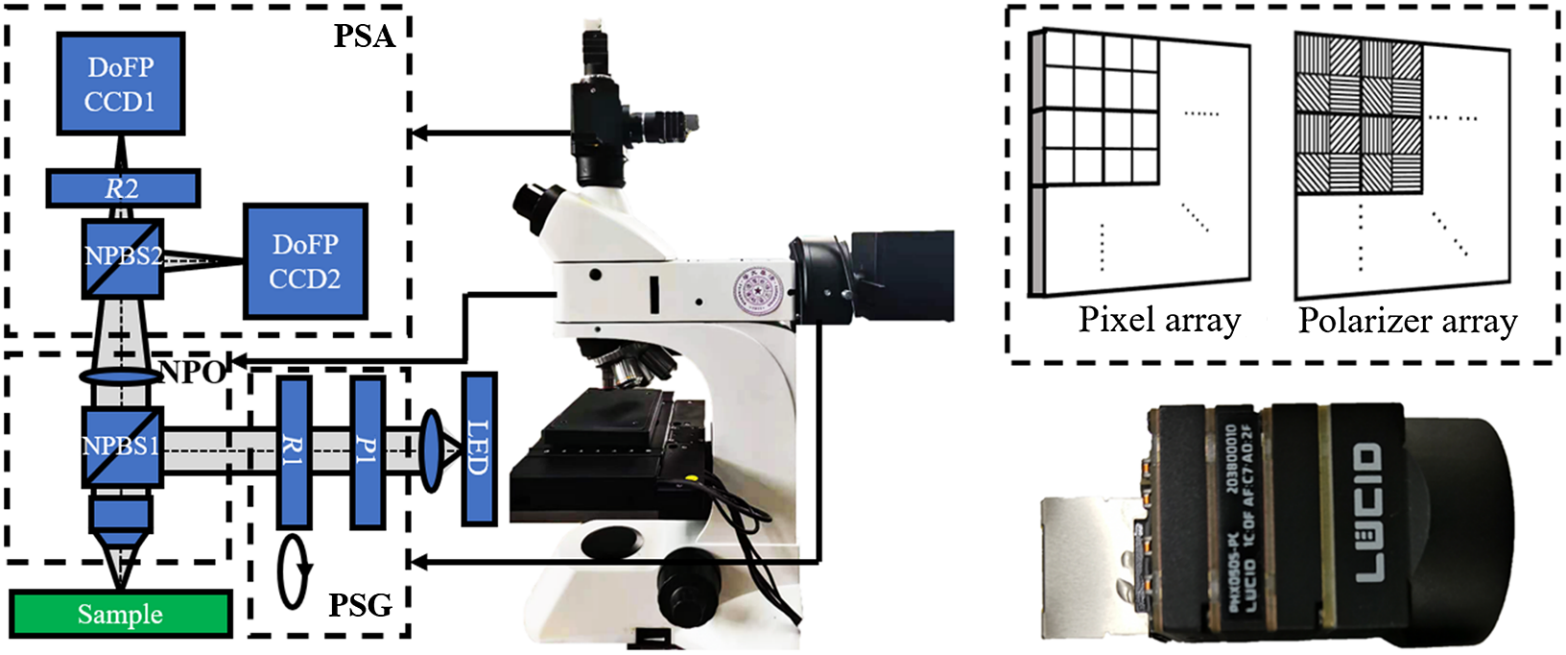
Schematic and photograph of (a) DoFPs-CRMMM. (b) DoFP polarimeter.

As shown in Fig. 1(a), the system is transformed by adding the PSG and the PSA module on a commercial metallurgical microscope (L3230, Guangzhou LISS Optical Instrument Co., Ltd., China). The epi-illumination is enabled by using a nonpolarized beam splitter NPBS1 (CCM1-BS013/M, Thorlabs Inc., United States) to ensure the illumination light path is collinear with the detection light path. The light emitted from the LED (633 nm, Δλ=20  nm) first undergoes polarization modulation by the PSG and is reflected by NPBS1, then focused by the objective lens (2810304, 4×, numerical aperture = 0.1, Guangzhou LISS Optical Instrument Co., Ltd., China) and illuminates the sample. The backscattered light from the sample passes through the objective lens, NPBS1 and the tube lens, then is detected by the PSA. The system is based on modular design: PSG module includes a fixed linear polarizer P1 (LPNIRE100-B, Thorlabs Inc., United States) and a rotatable quarter-wave plate R1 (WPQ10M-633, Thorlabs Inc., United States), PSA module includes two 16-bit DoFP polarimeters DoFP1 and DoFP2 (PHX050S-PC, Lucid Vision Labs Inc., Canada), a fixed quarter-wave plate R2 (WPQ10M-633, Thorlabs Inc., United States) and a 50:50 nonpolarized beam splitter NPBS2 (CCM1-BS013/M, Thorlabs Inc., United States). The objective lens, NPBS1, and tube lens together constitute a nonpolarization optics (NPO) module. Figure 1(b) shows the schematic of a single DoFP polarimeter, micropolarizers with four different polarization orientations are installed in front of every four adjacent pixels, which enables DoFP polarimeter to obtain four polarization channel images in a single shot.

We define DoFPs-CRMMM’s 0-deg direction as the horizontal x-axis from the vertical view of the microscope. In the PSA, DoFP1 and DoFP2 with the same resolution, field of view (FOV), and exposure time are fixed to NPBS2’s transmission end and reflection end, respectively, and are aligned with the 0-deg polarization orientation parallel to the 0-deg direction of DoFPs-CRMMM. The images acquired by two DoFP polarimeters have undergone bilinear interpolation and image registration in order to reduce possible polarization artifacts,^27^ the image registration is based on affine transformation, including reflection, translation, and image rotation, and mutual information (MI) is used as the criterion of registration accuracy. After the registration, image clipping is used to obtain the common area of the FOVs of two DoFP polarimeters. According to Ref. 25, to make the PSA optimized, R2 can be a quarter-wave plate simply fixed between NPBS2 and any of the DoFP polarimeters at any fast axis orientation. Without loss of generality, when R2 is fixed between NPBS2 and DoFP1 with the fast axis orientation parallel to the 0-deg direction of DoFPs-CRMMM, the 8×4 instrument matrix of the PSA can be calculated as

$$A_{\mathrm{PSA}}=[\begin{array}{l} A_{\mathrm{DoFP}}M_{R2}(0\;\deg,90\;\deg)M_{\mathrm{trans}\_\mathrm{NPBS}2}\\ A_{\mathrm{DoFP}}M_{\text{reflect}\_\mathrm{NPBS}2} \end{array}].$$ (1)

In $A_{\mathrm{PSA}}$, eight rows correspond to eight polarization analyzing channels of DoFP1 and DoFP2, which have highest sensitivity to specific polarization states. $M_{\mathrm{trans}\_\mathrm{NPBS}2}$ and $M_{\text{reflect}\_\mathrm{NPBS}2}$ are the MMs of NPBS2’s transmission end and reflection end, respectively. $A_{\mathrm{DoFP}}$ is the instrument matrix of DoFP polarimeter, when the micropolarizers in front of every four adjacent pixels in DoFP polarimeter have the polarization orientation of 0 deg, 45 deg, 90 deg,  and 135 deg, $A_{\mathrm{DoFP}}$ can be expressed as

$$A_{\mathrm{DoFP}}=0.5[\begin{array}{cccc} 1 & 1 & 0 & 0\\ 1 & 0 & 1 & 0\\ 1 & -1 & 0 & 0\\ 1 & 0 & -1 & 0 \end{array}].$$ (2)

In the PSG, the polarization orientation of P1 and the initial fast axis orientation of R1 are set to 0 deg and parallel to the 0-deg direction of DoFPs-CRMMM. During the MM measurement, R1’s fast axis orientation rotates n (n≥4) preset angles to generate four independent polarization states, then the Stokes vector of illuminated light can be calculated as

$$S_{\text{in}}=M_{R1}(\theta_{R1},90\;\deg)M_{P1}(0\;\deg)S_{\mathrm{LED}},\;\theta_{R1}\in \{\theta_{R1}(1),\ldots ,\theta_{R1}(n)\},$$ (3)

where $M_{R1}(\theta_{R1},90\;\deg)$ represents the MM of the quarter-wave plate R1 when the fast axis angle is $\theta_{R1}$, $M_{P1}(0\;\deg)$ represents the MM of the linear polarizer P1, and $S_{\mathrm{LED}}$ represents the polarization states of the LED’s illumination light. In this paper, n is set to 4 to reduce the measurement time of the MM, then the instrument matrix $A_{\mathrm{PSG}}$ of the PSG can be expressed as

$$A_{\mathrm{PSG}}=[S_{\text{in}}(1),S_{\text{in}}(2),S_{\text{in}}(3),S_{\text{in}}(4)]\;=[\begin{array}{cccc} 1 & 1 & 1 & 1\\ \cos^{2}\;2\theta_{R1}(1) & \cos^{2}\;2\theta_{R1}(2) & \cos^{2}\;2\theta_{R1}(3) & \cos^{2}\;2\theta_{R1}(4)\\ 0.5\;\sin\;4\theta_{R1}(1) & 0.5\;\sin\;4\theta_{R1}(2) & 0.5\;\sin\;4\theta_{R1}(3) & 0.5\;\sin\;4\theta_{R1}(4)\\ \sin\;2\theta_{R1}(1) & \sin\;2\theta_{R1}(2) & \sin\;2\theta_{R1}(3) & \sin\;2\theta_{R1}(4) \end{array}].$$ (4)

To reduce the influence of intensity measurement deviation, the condition number (CN) is used for evaluating the degree of the PSG’s optimization.^28^ According to Ref. 29, here we search the minimum CN of $A_{\mathrm{PSG}}$ using the genetic algorithm integrated in the MATLAB^®^ optimization toolbox, when R1’s fast axis orientations $\theta_{R1}$ are set to ±51.7  deg and ±15.1  deg, the CN of $A_{\mathrm{PSG}}$ reaches to the local minimum 3.40, which means the PSG is optimized.

Finally, sample’s MM can be calculated according to the equation

$$M_{\text{sample}}=A_{\mathrm{PSA}}^{-1}[I_{\text{sample}}]A_{\mathrm{PSG}}^{-1},$$ (5)

where $\left[I_{\text{sample}}\right]$ is an 8×4 matrix which contains intensity images corresponding to the four polarization orientations of DoFP1 and DoFP2 under four different $S_{\text{in}}$. The superscripts −1 represent the inverse or pseudoinverse of the matrix.

### 2.2 Improved Double-Pass Eigenvalue Calibration Method

Noted that there exist two systematic errors which may affect the measured MM. The first one is the parasitic polarization artifacts induced by the NPO module inside the microscope, including NPBS1, the objective lens, and the tube lens. The second one is the surface of NPO module may reflects light directly into the DoFP polarimeters without illuminating the sample, thus the measurement results will be interfered. Therefore, in the DoFPs-CRMMM, after taking the systematic errors from the NPO module into account, the measured output irradiance can be described by the following equation:

$$[I_{\text{sample}}]=i_{\mathrm{NPO}}+{A^{\prime }}_{\mathrm{PSA}}M_{\text{sample}}{A^{\prime }}_{\mathrm{PSG}},$$ (6)

the true instrument matrix of the PSG and the PSA is modified to

$${A^{\prime }}_{\mathrm{PSA}}=A_{\mathrm{PSA}}M_{\mathrm{trans}\_\mathrm{NPO}},$$ (7)

$${A^{\prime }}_{\mathrm{PSG}}=M_{\text{reflect}\_\mathrm{NPO}}A_{\mathrm{PSG}},$$ (8)

where $M_{\mathrm{trans}\_\mathrm{NPO}}$ and $M_{\text{reflect}\_\mathrm{NPO}}$ are the equivalent transmission and reflection MMs of the NPO module, $i_{\mathrm{NPO}}$ is the light directly reflected by the NPO module’s surface.

In the previous work, we demonstrated a transmission MM microscope based on dual DoFP polarimeters, the PSG and the PSA are calibrated separately.^25^ However, similar calibration method cannot be applied to DoFPs-CRMMM, since unknown $M_{\mathrm{trans}\_\mathrm{NPO}}$ and $M_{\text{reflect}\_\mathrm{NPO}}$ exist before and after light interacts with the sample, which change the true instrument matrices of the PSG and PSA. To calculate ${A^{\prime }}_{\mathrm{PSA}}$ and ${A^{\prime }}_{\mathrm{PSG}}$ and calibrate DoFPs-CRMMM, we improve the algorithm of the dp-ECM,^13^ which is a variant of eigenvalue calibration method^30^ and has been used for the calibration of MM confocal microscope. In comparison with the original dp-ECM, our method performs background light correction by considering the effect of directly reflected light from the NPO module’s surface, therefore is universal for the calibration of any collinear reflection MM measuring system. During the calibration, a mirror and some reference samples are measured. The steps are as follows:

(a) When using a mirror as the sample, the measured output irradiance can be expressed as
  $$[{I^{\prime }}_{\text{mirror}}]=[I_{\text{mirror}}-i_{\mathrm{NPO}}]={A^{\prime }}_{\mathrm{PSA}}M_{\text{mirror}}{A^{\prime }}_{\mathrm{PSG}},$$ (9)
  where $i_{\mathrm{NPO}}$ is measured by replacing the mirror with an optical absorption baffle (BF1, Thorlabs Inc., United States), the reflectance is about 1%. Ideal MM $M_{\text{mirror}}$ of the mirror under normal incidence is
  $$M_{\text{mirror}}=[\begin{array}{cccc} 1 & 0 & 0 & 0\\ 0 & 1 & 0 & 0\\ 0 & 0 & -1 & 0\\ 0 & 0 & 0 & -1 \end{array}].$$ (10)
(b) When measuring j’th reference samples on the mirror, the measured irradiance can be expressed as
  $$[{I^{\prime }}_{j}]=[I_{j}-(i_{\mathrm{NPO}}+i_{j})]={A^{\prime }}_{\mathrm{PSA}}{M^{\prime }}_{j}{A^{\prime }}_{\mathrm{PSG}},$$ (11)
  here, we adopt polarization optics including linear polarizer and phase retarder as the reference samples, since the light passes twice through the reference sample, the MM of the combination of the reference sample and the mirror can be expressed as
  $${M^{\prime }}_{j}=M_{j}(\theta )M_{\text{mirror}}M_{j}(-\theta ),$$ (12)
  and because reference samples usually have a smooth surface that also reflects light $i_{j}$ directly, which will cause a portion of light detected by PSA without undergoing the polarization modulation of the reference sample and affect the calibration performance, thus $\left(i_{\mathrm{NPO}}+i_{j}\right)$ must be subtracted from the measured irradiance, $\left(i_{\mathrm{NPO}}+i_{j}\right)$ can be derived by measuring the reference sample on an optical absorption baffle.
(c) Based on the results from (a) and (b), we can get the matrices
  $$C_{j}={\left[{I^{\prime }}_{\text{mirror}}\right]}^{-1}[{I^{\prime }}_{j}]={A^{\prime }}_{\mathrm{PSG}}^{-1}M_{\text{mirror}}^{-1}{M^{\prime }}_{j}{A^{\prime }}_{\mathrm{PSG}}.$$ (13)
(d) The true instrument matrix ${A^{\prime }}_{\mathrm{PSG}}$ of PSG can be determined by solving the equation $H_{j}$
  $$H_{j}=M_{\text{mirror}}^{-1}{M^{\prime }}_{j}{A^{\prime }}_{\mathrm{PSG}}-{A^{\prime }}_{\mathrm{PSG}}C_{j}=0,$$ (14)
  to solve the equation, we construct the matrix K composed of $H_{j}$
  $$K=\underset{j}{\sum }H_{j}^{T}H_{j},$$ (15)
  where K is a positive symmetric real matrix with 15 non-null and 1 null eigenvalues, since ${A^{\prime }}_{\mathrm{PSG}}$ is the only solution of Eq. (14), ${A^{\prime }}_{\mathrm{PSG}}$ is the eigenvector of K corresponding to the smallest eigenvalue $\lambda_{16}$ equals or closest to 0. After ${A^{\prime }}_{\mathrm{PSG}}$ is determined, ${A^{\prime }}_{\mathrm{PSA}}$ can be calculated as
  $${A^{\prime }}_{\mathrm{PSA}}=[{I^{\prime }}_{\text{mirror}}]{A^{\prime }}_{\mathrm{PSG}}^{-1}M_{\text{mirror}}^{-1}.$$ (16)
(e) Once ${A^{\prime }}_{\mathrm{PSG}}$ and ${A^{\prime }}_{\mathrm{PSA}}$ are both determined, the MM of the sample can be calculated according to the equation
  $$M_{\text{sample}}={A^{\prime }}_{\mathrm{PSA}}^{-1}[I_{\text{sample}}-i_{\mathrm{NPO}}]{A^{\prime }}_{\mathrm{PSG}}^{-1}.$$ (17)

**Fig. 2:**
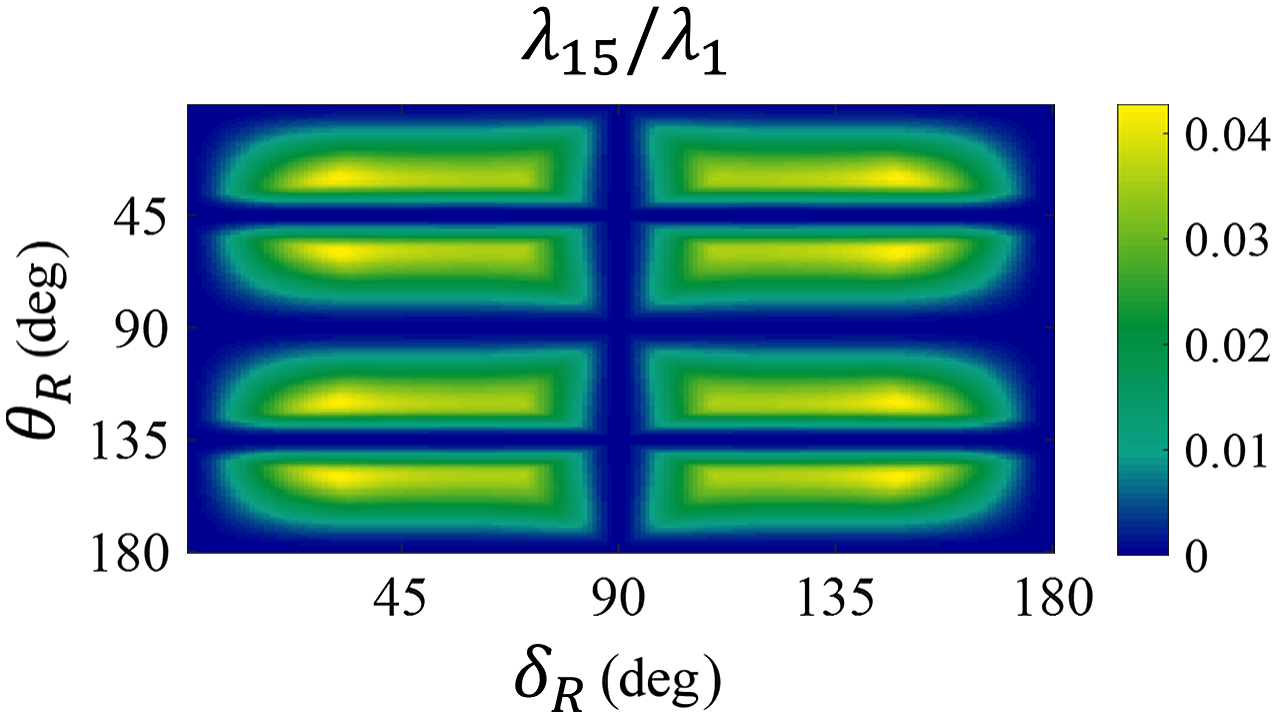
The relationship between $\lambda_{15}/\lambda_{1}$, $\theta_{R}$, and $\delta_{R}$.

To ensure Eq. (14) has a unique solution as ${A^{\prime }}_{\mathrm{PSG}}$, several reference samples are needed so that the dimension of null space of K can be reduced to 1. According to Ref. 30, the best-chosen set of reference samples should satisfy that the non-null eigenvalue $\lambda_{15}/\lambda_{1}$ of K reaches maximum, which enables ${A^{\prime }}_{\mathrm{PSG}}$ to be determined with best precision. For transmission or noncollinear MM measuring systems which adopts single-pass ECM (sp-ECM) for calibration, two linear polarizers (the polarization orientations are horizontal and vertical, respectively) and a quarter-wave plate aligned at around 30 deg is optimal.^13^^,^^30^ However, in the collinear reflection MM measuring system, since the double pass MM of the quarter-wave plate is close to the mirror, K would have two eigenvalue close to 0, which makes Eq. (14) has no unique solution and cause calibration failure. Here, we adopt three reference samples: two linear polarizers with 0 deg and 90 deg polarization orientation, respectively, and a phase retarder with the fast axis orientation $\theta_{R}$ and the retardance $\delta_{R}$. As shown in Fig. 2, $\theta_{R}$ equals to 0 deg or the multiples of 45 deg and $\delta_{R}$ equals to 0 deg or the multiples of 90 deg should be avoided since $\lambda_{15}/\lambda_{1}$ is close to 0, which means Eq. (14) is unsolvable, thus in collinear reflection scheme, quarter-wave plate using for operating wavelength must be avoided, however, as shown in Fig. 2, quarter-wave plate using for some other nonoperating wavelengths or wave plate with other retardance (25 deg to 75 deg) is accecptable. Here, we use a 1/8 wave plate (OWP-633, Shenzhen LUBANG Technology Co., Ltd., China), when $\theta_{R}$ equals to 30.6 deg, $\lambda_{15}/\lambda_{1}$ reaches the local maximum which provides the optimal accuracy for the calibration.

To evaluate the measurement performance of DoFPs-CRMMM after calibration, we measure the MM of the standard polarization samples using DoFPs-CRMMM to verify the measurement accuracy and speed. The standard polarization samples including a linear polarizer (FLP20-VIS, Shenzhen LUBANG Technology Co., Ltd., China) on the mirror and a wave plate (OWP-633, Shenzhen LUBANG Technology Co., Ltd., China) of 46.3-deg retardance on the mirror, the retardance of the standard wave plate was measured by a transmission MM microscope before the experiment. The linear polarizer and the wave plate are assumed to be ideal, then the true values are derived by caculating the MMs of the standard polarization samples under different azimuths. Before the measurement, we use three calibration methods to calibrate DoFPs-CRMMM and compare their calibration performances. In method a, the system is calibrated by the improved dp-ECM described in Sec. 2.2 using the 1/8 wave plate as one of the reference samples, and perform background light correction. In method b, the dp-ECM is carried out using the quarter wave plate as one of the reference samples and perform background light correction. In method c, the system is calibrated by the dp-ECM using the 1/8 wave plate as one of the reference samples, but without background light correction. Besides, in order to quantify the measurement accuracy of DoFPs-CRMMM after different calibration methods, the mean absolute error (MAE) of the measured MMs of the linear polarizer and the wave plate on the mirror is calculated according to Eq. (18). In order to quantify the noise level of DoFPs-CRMMM after different calibration methods, the standard deviation of the measured MM element images is calculated according to Eq. (19), where N stands for the pixel number of a MM element image. We also calculate the average value of MAE and std of the standard polarization samples under different azimuths for a comprehensive evaluation, and name them as $\overset{¯}{\mathrm{MAE}}$ and $\overset{¯}{\mathrm{std}}$, respectively

$$\mathrm{MAE}=\frac{1}{16}\overset{16}{\underset{n=1}{\sum }}|{\text{measured}}_{n}-{\text{true}}_{n}|.$$ (18)

$$\mathrm{std}=\frac{1}{16}\overset{16}{\underset{n=1}{\sum }}(\sqrt{\frac{1}{N-1}\overset{N}{\underset{i=1}{\sum }}{\left(\text{measured}-\text{average}(\text{measured})\right)}^{2}}).$$ (19)

## 3 Experimental Results

### 3.1 Validation Experiment

**Fig. 3:**
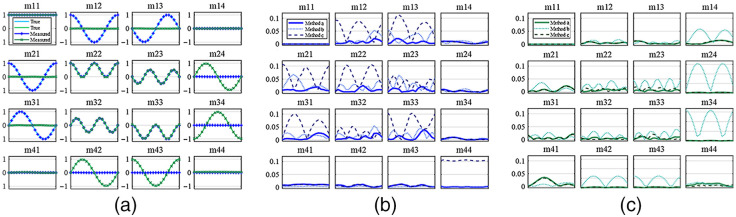
(a) MMs of the linear polarizer (blue) and wave plate (green) with different azimuths on the mirror measured by DoFPs-CRMMM using method a. (b) The absolute errors of MMs of the linear polarizer with different azimuths on the mirror measured by DoFPs-CRMMM using different calibration methods. (c) The absolute errors of MMs of the wave plate with different azimuths on the mirror measured by DoFPs-CRMMM using different calibration methods. (method a: improved dp-ECM using a 1/8 wave plate and background light correction; method b: dp-ECM using a quarter wave plate and background light correction; method c: dp-ECM using a 1/8 wave plate without background light correction). The display range of m24, m34, m42, and m43 in (c) is [0, 1.68] for better visualization.

Figure 3 gives the results of two standard polarization samples measured by DoFPs-CRMMM under different calibration methods. The azimuth of the standard polarization samples varies from 0 deg to 170 deg in 10 deg steps. Figure 3(a) shows the MMs of two standard polarization samples after calibration using the improved dp-ECM, the MMs are derived by calculating the average values of MM element images, the results indicate after the calibration, the measured MMs are very close to the true values. We also calculate the absolute errors based on the true values of standard polarization samples. As shown in Figs. 3(b) and 3(c), the absolute errors of MMs indicate that the calibration accuracy of the improved dp-ECM is higher than other two calibration method on two types of the standard polarization samples on the overall level. In comparison, calibration methods b and c cannot achieve good calibration performance on all the standard polarization samples, and the systematic errors periodically affect different MM elements.

Table 1 shows the results of the quantitative evaluation of DoFPs-CRMMM’s measurement performance on MMs after calibration, including $\overset{¯}{\mathrm{MAE}}$ and $\overset{¯}{\mathrm{std}}$. As for the measurement accuracy of DoFPs-CRMMM, the results in Table 1 indicate the method a has the smallest $\overset{¯}{\mathrm{MAE}}$ of less than 1% on both linear polarizer and wave plate, which means the calibration method a has satisfactory calibration accuracy. As for the calibration method b, when using the quarter-wave plate rather than the 1/8 wave plate as the reference sample in dp-ECM, the $\overset{¯}{\mathrm{MAE}}$ of the linear polarizer has doubled to about 1.3%, and when comes to the wave plate, the $\overset{¯}{\mathrm{MAE}}$ has greatly increased to about 19% mainly due to the huge deviations in m24, m34, m42, and m43, which proves the necessity of optimizing the reference samples set in ECM. As for the calibration method c, $\overset{¯}{\mathrm{MAE}}$ of the standard polarization samples increased because of the effect of the directly reflected light from the surface of NPO module and the reference samples, and the MMs of linear polarizer are particularly vulnerable. In addition, the results also show the significance of measuring multiple kinds of standard polarization samples to characterize the calibration accuracy of the calibration method.

**Table 1:** Average MAE and average standard deviation of the MM elements after three different calibration methods.

	$\overset{¯}{\mathrm{MAE}}$		$\overset{¯}{\mathrm{std}}$	
	Linear polarizer and mirror	Wave plate and mirror	Linear polarizer and mirror	Wave plate and mirror
Method a^a^	0.0070	0.0097	0.0048	0.0045
Method b^b^	0.0134	0.1857	0.0048	0.0483
Method c^c^	0.0351	0.0108	0.0040	0.0044

a Method a: dp-ECM using a 1/8 wave plate and background light correction.

b Method b: dp-ECM using a quarter wave plate and background light correction.

c Method c: dp-ECM using a 1/8 wave plate without background light correction.

As for the noise level of DoFPs-CRMMM, $\overset{¯}{\mathrm{std}}$ represents the spatial variation of the MM element images result from the statical noise and the spatial fluctuation of the standard polarization samples. Since the imaging position on the sample remains unchanged between three calibration methods, the relative magnitude of the noise level of three calibration methods can be compared by $\overset{¯}{\mathrm{std}}$. The results in Table 1 shows after calibration using method a, DoFPs-CRMMM can achieve a low noise level that $\overset{¯}{\mathrm{std}}<0.5\%$. Calibration methods b and c show similar results, except for the wave plate calibrated by method b, the rise of noise level is because the MM measurement noise is amplified due to the unoptimized set of reference samples.

**Fig. 4:**
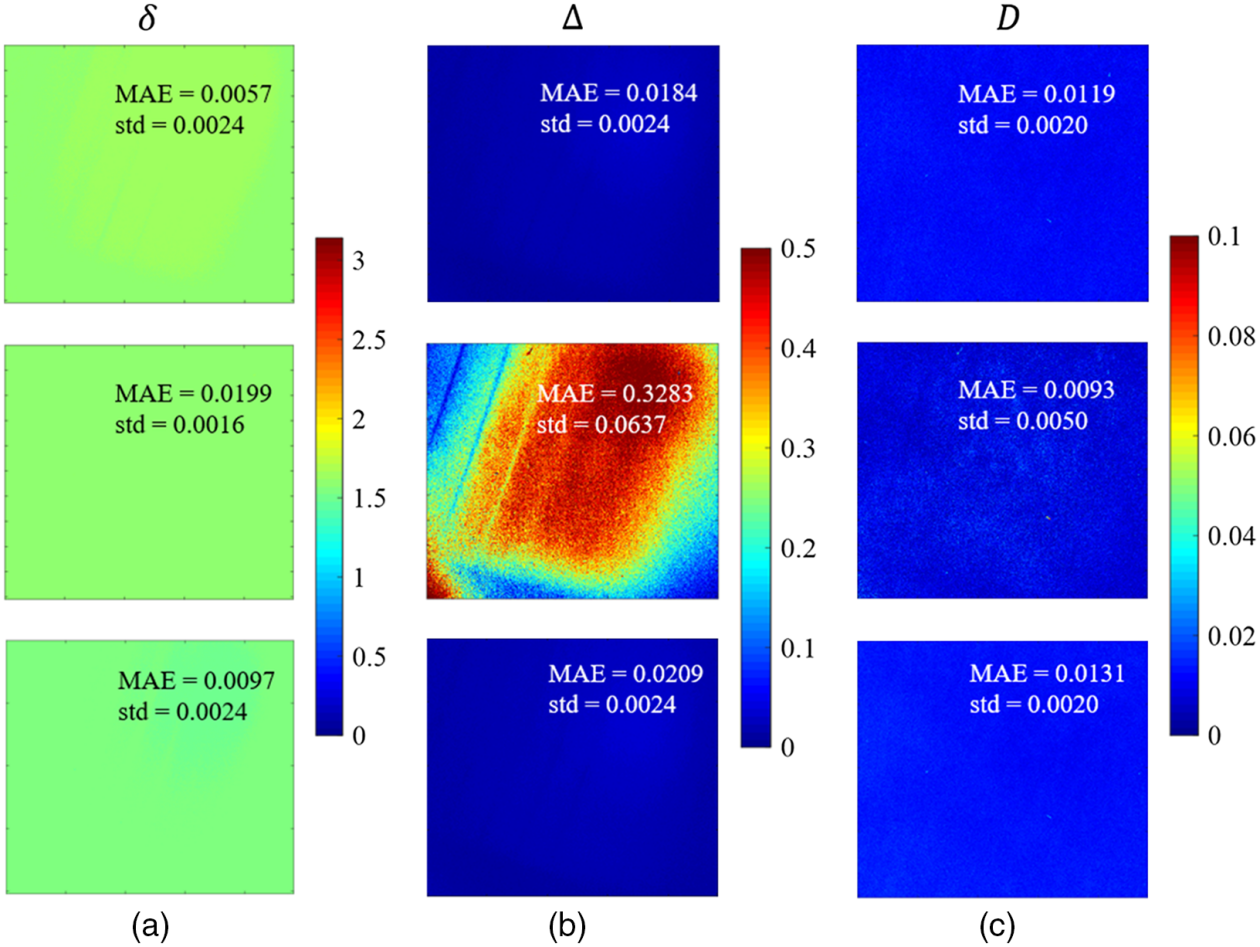
MMD parameters (a) linear retardance δ, (b) depolarization Δ and (c) diattenuation D of the wave plate with 0 deg azimuth on the mirror calibrated by method a (the first row), method b (the second row), and method c (the third row).

MM transforms the polarization states. It contains rich information on the properties of the sample but lacks clear physical meanings. To evaluate the measurement accuracy of polarization parameters explicitly related to the physical properties, we calculate Mueller matrix decomposition (MMD) parameters^31^ of the measured wave plate on the mirror including the linear retardance δ, depolarization Δ and diattenuation D. The images of MMD parameters of the measured wave plate with 0-deg azimuth on the mirror calibrated by methods a to c are shown in Fig. 4. The results indicate that depolarization and diattenuation parameters calibrated by method b show visible spatial nonuniform due to the poor calibration performance. To quantitatively evaluate the measurement performance on standard sample, since an ideal wave plate only has the property of retardance, and the depolarization and diattenuation parameters are close to 0, we calculate MAE according to the polarization parameter image’s average value, and the standard deviation is calculated based on the 500×500  pixels area in the upper right of the FOV to reduce the impact of the sample’s spatial nonuniformity. Results show after calibrated by method a using background light correction and reference samples set optimization, polarization parameters have slightly lower MAE and standard deviation than method c. The polarization parameters measured by DoFPs-CRMMM have high measurement quality, which is important in practical application.

The measurement speed is critical in MM monitoring of dynamic process. There are several parameters closely related to the measurement speed including the number of acquisitions, the range of the wave plate rotation angles, and the speed of rotation. As for the acquisition numbers, DRR-CRMMM need 30 acquisitions to calculate the Fourier coefficients and reconstruct MM, while DoFPs-CRMMM only needs to rotate R1 to four angles and DoFP polarimeters perform four acquisitions to calculate MM. Besides, the range of total rotation angles for R1 and R2 in DRR-CRMMM are 180 deg and 900 deg, respectively, while the range of total rotation angles for R1 in DoFPs-CRMMM is only 103.4 deg. DoFPs-CRMMM needs smaller acquisition numbers and smaller rotation range for the wave plate than DRR-CRMMM, which means using the same speed of the rotation stage, DoFPs-CRMMM is capable of much faster MM measurements. In addition, DoFPs-CRMMM adopts a high-speed rotation stage (DDR25/M, Thorlabs Inc., United States) with the maximum rotation speed of up to 5 Hz,. During the validation experiment, the exposure time of DoFP polarimeters is set to 0.3 ms, and the frames per second (FPS) is set to 20 Hz, the acquisition time of a single MM is about 2.6 s. Since the dual DoFP polarimeters based PSA is capable of detecting polarization states in a single shot, the measurement speed of DoFPs-CRMMM is mainly determined by the rotating retarder-based PSG, therefore, the MM imaging speed of the current system can be further increased using faster modulation in PSG such as a faster-rotating wave plate, or a liquid-crystal variable retarder (LCVR).^32^ Ideally, when PSG and PSA are synchronized using trigger signal, DoFPs-CRMMM can calculate one MM from four exposures, so as to realize near-real-time MM microscopic imaging.

**Fig. 5:**
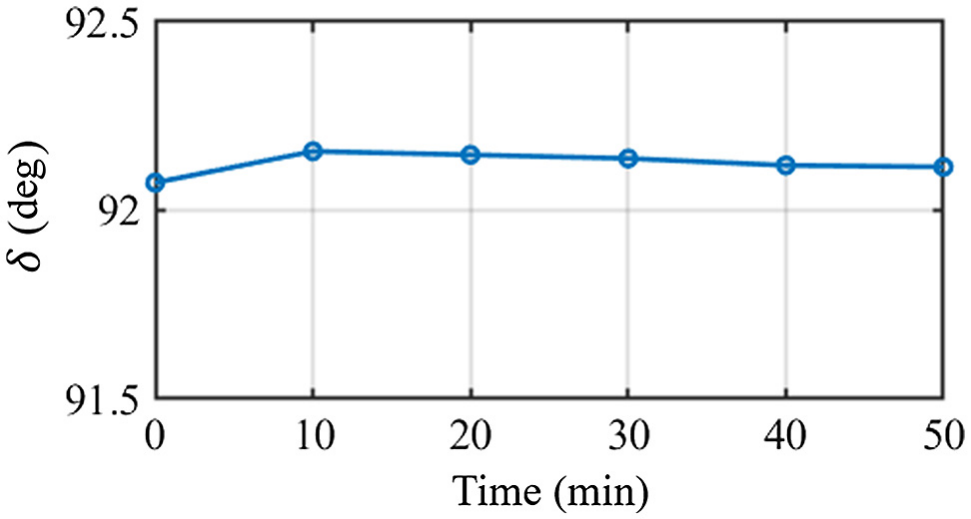
Measured retardance of the wave plate on the mirror during 1-h measurement.

Since the aim of DoFPs-CRMMM is for dynamic process monitoring, system’s temporal stability is of great importance. To validate the system’s temporal stability, DoFPs-CRMMM is used to measure the wave plate on the mirror every 10 minutes for 1 h, the average value of linear retardance δ during the measurement are calculated and shown in Fig. 5. The standard deviation is <0.03  deg. The results show that DoFPs-CRMMM has high temporal stability, making the system a potential tool for monitoring long-time dynamic process accurately.

### 3.2 Using the DoFPs-CRMMM for Dynamic Process Monitoring

**Fig. 6:**
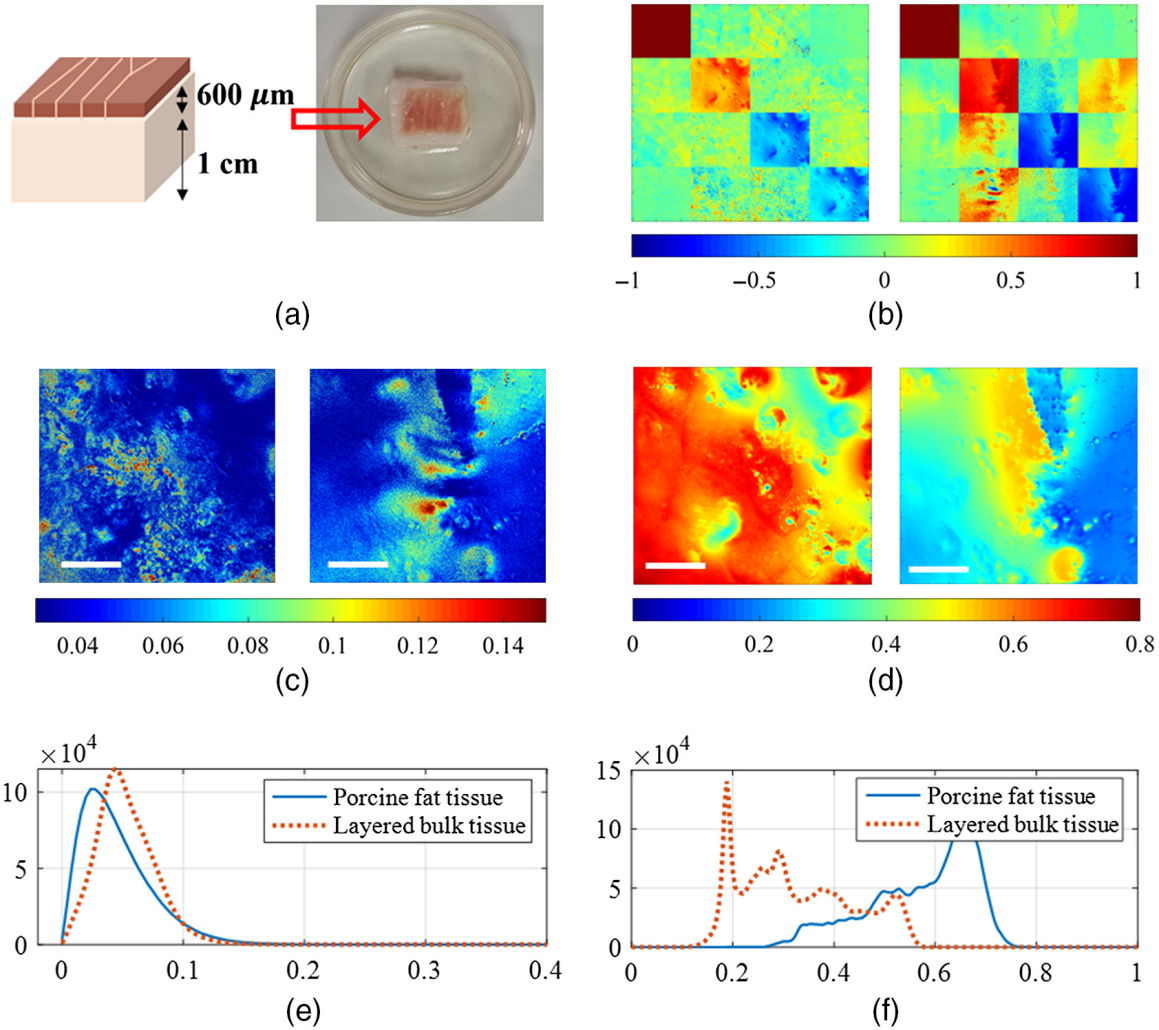
(a) Schematic and photograph of the layered bulk tissue. (b) MM images of the porcine fat tissue (left) and the layered bulk tissue (right). (c) Images and (e) histogram of linear retardance δ of the porcine fat tissue (left, blue solid line) and the layered bulk tissue (right, red dotted line). (d) Images and (f) histogram of depolarization D of the porcine fat tissue (left, blue solid line) and the layered bulk tissue (right, red dotted line). The length of the scale bar is 100  μm.

**Fig. 7:**
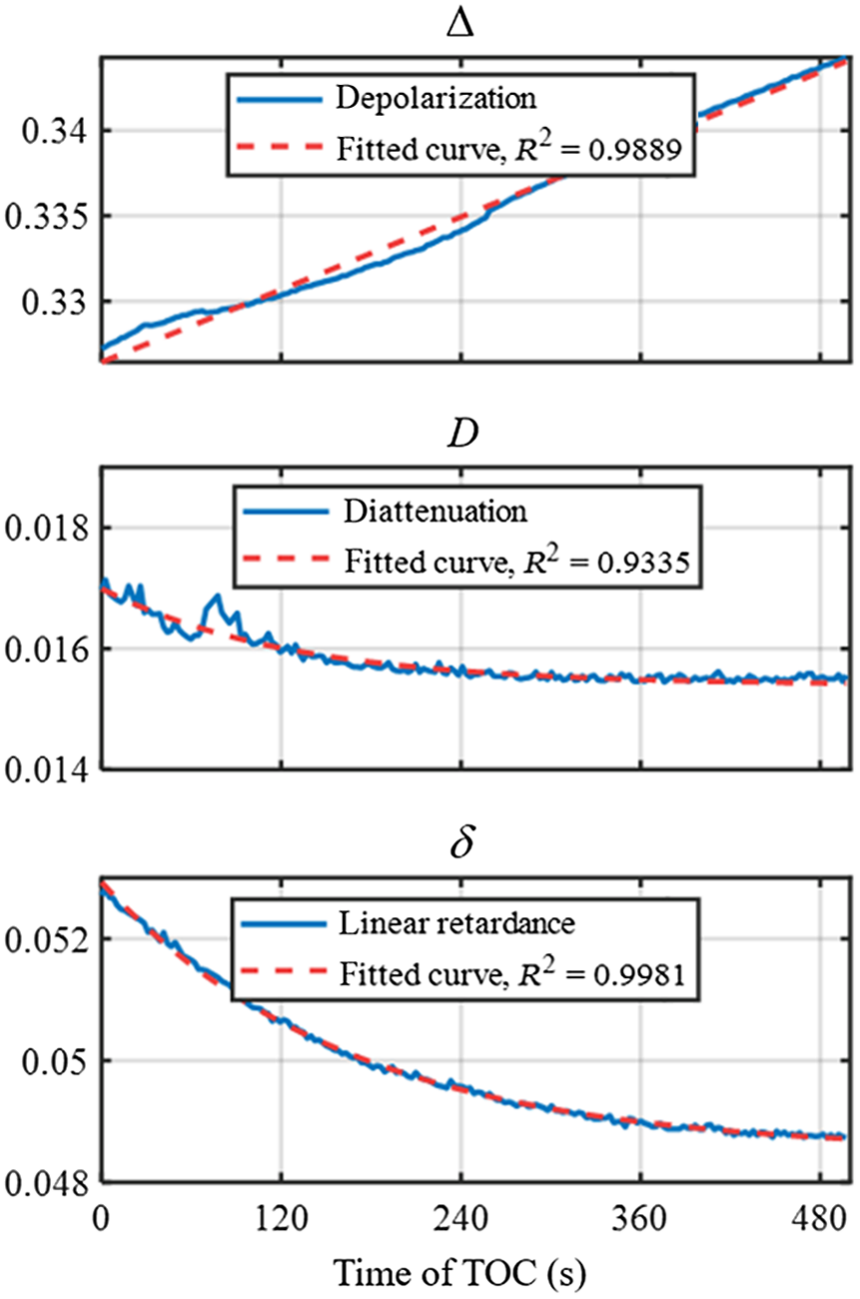
Average values of the layered bulk tissue’s MMD parameters during TOC process.

In this experiment, DoFPs-CRMMM is tested for probing dynamic process. We use a vibrating blade microtome (VT1200 S, Leica Biosystems, Germany) to cut a 600-μm thickness bovine skeletal muscle slice and place it over a 1-cm thickness porcine fat to prepare a layered bulk tissue, as shown in Fig. 6(a). MM images of the porcine fat tissue and the layered bulk tissue are also measured, as shown in Fig. 6(b), where the display range of nondiagonal elements is set to [−0.1  0.1] for better visualization. We also calculate the MMD parameters to represent the polarization properties. The images and the histogram of the MMD parameters are also shown in Fig. 6. The results show the porcine fat tissue is mostly isotropic with relatively large depolarization, and the layered bulk tissue (with the bovine skeletal muscle as the upper layer) shows prominent anisotropic features in retardance with smaller depolarization. Then DoFPs-CRMMM is applied to the polarization monitoring of the layered bulk tissue during TOC process. We drop 100% glycerol solution (G116203, Shanghai Aladdin Biochemical Technology Co., Ltd., China) on the tissue and wait until the solution penetrates totally, then DoFPs-CRMMM is used to measure continuously for 500 s, and 193 MM images are acquired. MMD parameters are also derived to better characterize the polarization properties. Figure 7(a) displays the change of average values of MMD parameters during TOC process. We fit curves for the corresponding MMD parameters using linear regression model (depolarization Δ) and exponential regression model (diattenuation D and linear retardance δ), and calculate the coefficient of determination $R^{2}$ using the MATLAB^®^ curve fitting toolbox. $R^{2}$ of the fittings are over 90%, indicating good descriptions for the variation trend of polarization parameters. Table 2 shows the coefficients and 95% confidence intervals of the fitted curves. The fitted results show that as the TOC progresses, different polarization parameters behave differently during the TOC process. Depolarization varies linearly with time, but dichroism and retardance vary exponentially with different time constants, 124 and 150 s, respectively, which indicates the change of depolarization is a longer process, and other two properties’ variation mainly happens in the first few minutes. The changes of the layered bulk tissue’s polarization properties may attribute to as the bovine skeletal muscle slice becomes transparent gradually, the detection depth and the proportion of the signal from the porcine fat increase. The results show DoFPs-CRMMM has the ability to capture polarization properties of dynamic process in a small time resolution.

**Table 2:** Coefficients and 95% confidence intervals of the fitted curve of MMD parameters obtained with different acquisition times.

	Δ: f(x)=ax+b		
τ	a	b	
2.6 s	$3.542\times 10^{-5}$ $\left[3.488\times 10^{-5},3.597\times 10^{-5}\right]$	0.3264	
10.4 s	$3.542\times 10^{-5}$ $\left[3.443\times 10^{-5},3.650\times 10^{-5}\right]$	0.3264	
20.8 s	$3.563\times 10^{-5}$ $\left[3.412\times 10^{-5},3.714\times 10^{-5}\right]$	0.3262	
31.2 s	$3.584\times 10^{-5}$ $\left[3.393\times 10^{-5},3.776\times 10^{-5}\right]$	0.3261	
41.6 s	$3.574\times 10^{-5}$ $\left[3.340\times 10^{-5},3.807\times 10^{-5}\right]$	0.3260	
	D: $f(x)=ae^{bx}+c$		
τ	a	b	c
2.6 s	$15.94\times 10^{-4}$ $\left[15.33\times 10^{-4},16.55\times 10^{-4}\right]$	$-79.79\times 10^{-4}$ $\left[-84.09\times 10^{-4},-75.49\times 10^{-4}\right]$	0.0154
10.4 s	$43.06\times 10^{-4}$ $\left[39.24\times 10^{-4},46.87\times 10^{-4}\right]$	$-72.63\times 10^{-4}$ $\left[-81.92\times 10^{-4},-63.33\times 10^{-4}\right]$	0.0154
20.8 s	$65.85\times 10^{-4}$ $\left[59.74\times 10^{-4},71.95\times 10^{-4}\right]$	$-54.98\times 10^{-4}$ $\left[-62.58\times 10^{-4},-47.39\times 10^{-4}\right]$	0.0153
31.2 s	$86.43\times 10^{-4}$ $\left[79.48\times 10^{-4},93.38\times 10^{-4}\right]$	$-47.40\times 10^{-4}$ $\left[-53.24\times 10^{-4},-41.56\times 10^{-4}\right]$	0.0153
41.6 s	$107.6\times 10^{-4}$ $\left[98.62\times 10^{-4},116.6\times 10^{-4}\right]$	$-43.30\times 10^{-4}$ $\left[-48.97\times 10^{-4},-37.63\times 10^{-4}\right]$	0.0165
	δ: $f(x)=ae^{bx}+c$		
τ	a	b	c
2.6 s	$44.28\times 10^{-4}$ $\left[44.02\times 10^{-4},44.53\times 10^{-4}\right]$	$-61.01\times 10^{-4}$ $\left[-61.51\times 10^{-4},-60.50\times 10^{-4}\right]$	0.0485
10.4 s	$46.82\times 10^{-4}$ $\left[45.99\times 10^{-4},47.64\times 10^{-4}\right]$	$-57.28\times 10^{-4}$ $\left[-58.78\times 10^{-4},-55.79\times 10^{-4}\right]$	0.0485
20.8 s	$50.26\times 10^{-4}$ $\left[48.45\times 10^{-4},52.07\times 10^{-4}\right]$	$-53.16\times 10^{-4}$ $\left[-56.03\times 10^{-4},-50.30\times 10^{-4}\right]$	0.0485
31.2 s	$54.20\times 10^{-4}$ $\left[50.19\times 10^{-4},58.21\times 10^{-4}\right]$	$-50.03\times 10^{-4}$ $\left[-55.64\times 10^{-4},-44.43\times 10^{-4}\right]$	0.0485
41.6 s	$56.60\times 10^{-4}$ $\left[49.55\times 10^{-4},63.64\times 10^{-4}\right]$	$-45.54\times 10^{-4}$ $\left[-54.31\times 10^{-4},-36.77\times 10^{-4}\right]$	0.0485

In addition, we also study the effect of MM’s measurement speed on measurement accuracy of polarization properties during the TOC process. Through sampling the original data of output Stokes vectors at different sampling rates, MMs under different acquisition time τ (2.6, 10.4, 20.8, 31.2, and 41.6 s) are calculated, then the fitted curves of the corresponding MMD parameters are calculated according to linear equation and exponential equation, respectively. Table 2 shows the coefficients and 95% confidence intervals of the fitted curve of MMD parameters derived at different sampling rates. During the TOC process, with the increase of MM acquisition time, the coefficients of the fitted curves of the polarization parameters changes, which means errors are introduced to the measured polarization parameters of dynamic process. This mainly due to the mismatch between the time resolution of the sample’s polarization properties variation and the time resolution of MM measurement system. The results prove the importance of MM’s measurement speed in dynamic process application, which is very common in living specimen, and much faster speed has to be considered when MM imaging.

## 4 Conclusion

We report a design named DoFPs-CRMMM for fast collinear reflection MM imaging. The improved dp-ECM is performed for system calibration to eliminate the effect of systematic errors. The system’s performances including measurement speed, measurement accuracy, and temporal stability are validated by measuring standard polarization samples. Preliminary results of monitoring the microstructural variation of artificial layered bulk tissues during TOC show that the system can be used for structure characterization during dynamic process, and that errors in polarization measurement are very sensitive to measurement time, which provides the motivation for developing faster or snapshot polarization imaging techniques.

## Code, Data, and Materials Availability

Data underlying the results presented in this paper are not publicly available at this time but may be obtained from the authors upon reasonable request.
